# Primary Clear Cell Adenocarcinoma of the Cervix: A Series of 12 Cases Without Exposure to Diethylstilbestrol

**DOI:** 10.7759/cureus.90543

**Published:** 2025-08-20

**Authors:** Aqueel Shahid, Tabinda Sadaf, Muhammad Anas Tehseen Asar, Sadia Anjum, Asma Rashid, Anusha Haider, Fajar Rafi Ranjha

**Affiliations:** 1 Clinical and Radiation Oncology, Shaukat Khanum Memorial Cancer Hospital and Research Centre, Lahore, PAK

**Keywords:** cervical cancer, clear cell adenocarcinoma, concurrent chemoradiotherapy, diethylstilbestrol (des)-unexposed, rare cervical malignancy

## Abstract

Background and objective

Clear cell adenocarcinoma of the uterine cervix (CCAUC) is a rare malignancy, historically linked to in utero diethylstilbestrol (DES) exposure. However, several cases have been reported in non-DES-exposed populations, particularly in Asia. This study aimed to evaluate the clinicopathological features, treatment modalities, and outcomes in patients with CCAUC unassociated with DES exposure.

Methods

We conducted a retrospective analysis of 12 patients diagnosed with CCAUC between 2015 and 2022 at Shaukat Khanum Memorial Cancer Hospital, Lahore, Pakistan. All diagnoses were confirmed by central pathology review. Patient data included demographics, clinical presentation, staging (FIGO: International Federation of Gynecology and Obstetrics), treatment details, and outcomes. Treatment approaches varied by disease stage and included surgery, chemotherapy, and concurrent chemoradiotherapy (CCRT).

Results

The mean age at diagnosis was 46.25 years, with most patients being premenopausal. Tumor size exceeded 4 cm in 83.3% of cases, with 50% cases being either stage FIGO IIB or IIIA. A majority (91.7%) received CCRT. The five-year overall survival was 64.3%, and disease recurrence occurred in 25% of patients, with distant metastases being the most common.

Conclusions

CCAUC in non-DES-exposed women tends to manifest in older age groups with bulky disease. Although treatment strategies are not standardized, primary surgery remains favorable for early-stage disease, while CCRT is appropriate for advanced stages. Given the rarity and poor prognosis of CCAUC, larger prospective studies are warranted to refine treatment guidelines and improve outcomes.

## Introduction

Cervical cancer remains the fourth most common malignancy in females globally [1], with significant health implications. While squamous cell carcinomas (SCCs) predominantly arise from the ectocervix, adenocarcinomas are more common in the endocervix [2]. Clear cell adenocarcinoma of the uterine cervix (CCAUC) is very rare, accounting for only 4-9% of all cervical adenocarcinomas [3]. Historically, CCAUC was associated with in utero exposure to diethylstilbestrol (DES), a synthetic estrogen. However, it has since been established that CCAUC can occur independently of DES exposure [4]. Larger studies have demonstrated that high-risk human papillomavirus (HPV), a known etiologic agent in most cervical cancers, is unlikely to play a major role in CCAUC pathogenesis [5]. Other suspected risk factors include cervical endometriosis, oral contraceptive use, and HIV infection.

In Jiang et al.’s study [6], which focused on non-DES-exposed patients, a bimodal age distribution was noted, with a median age of 38 years. Conversely, other studies have reported median ages of 52-53 years, indicating a higher prevalence in postmenopausal women [7,8]. Vaginal bleeding is a common presentation of CCAUC [9]. Given the rarity of CCAUC, especially in Asian populations without DES exposure, there is scarce data regarding its clinical behavior, pathology, treatment protocols, and prognosis. This study presents an analysis of 12 such cases to elucidate clinicopathologic characteristics and treatment outcomes, aiming to contribute to more effective clinical decision-making.

## Materials and methods

We retrospectively analyzed 12 patients diagnosed with CCAUC and treated between 2015 and 2022 at the Department of Clinical and Radiation Oncology, Shaukat Khanum Memorial Cancer Hospital, Lahore. All cases were confirmed via central pathology review. Clinical staging was performed on the basis of the FIGO (International Federation of Gynecology and Obstetrics) cervical cancer staging system 2018. Patients treated between 2015 to 2017 were restaged according to the FIGO staging system 2018 during analysis. Data collected included demographic details (age, marital and menstrual history), presenting symptoms, histologic and immunohistochemical findings, treatment modalities, and outcomes.

Surgical interventions included radical hysterectomy with bilateral salpingo-oophorectomy and pelvic lymphadenectomy, with or without para-aortic dissection. Adjuvant therapy (chemotherapy or radiotherapy) or radical chemoradiation was administered based on tumor stage, risk stratification, and multidisciplinary tumor board recommendations. All patients treated with radical intent were planned with image-guided radiotherapy (IGRT) technique, while the one patient treated in palliative intent was planned using the 3D conformal technique. Planning target volume (PTV) coverage and organs at risk (OARs) dose constraints were based on hospital protocol. Patients undergoing concurrent chemotherapy were either given cisplatin 40mg/m^2^ weekly or carboplatin AUC twice weekly, depending upon their kidney function. Exclusion criteria included patients with prior malignancy or those who died due to non-cancer-related causes. Frequency analysis of the data was performed using IBM SPSS Statistics version 20.0 (IBM Corp., Armonk, NY).

## Results

The mean age at diagnosis was 46.25 ± 17.44 years, with a peak incidence observed between 45 and 60 years. Most patients (66.7%) were premenopausal. Tumor size exceeded 4 cm in 10 patients (83.3%), indicating a predominance of bulky disease at presentation. According to the FIGO clinical staging system, two patients (16.7%) were diagnosed at stage IIA, three (25%) at stage IIB, and three (25%) at stage IIIA (Table 1).

**Table 1:** Clinicopathological features Carb/Pac: carboplatin + paclitaxel; FIGO: International Federation of Gynecology and Obstetrics; SD: standard deviation

Variables	Values
Age, years, mean, ± SD	46.25 17.44
Menopausal status, n (%)	
Pre- menopausal	8 (66.7%)
Post- menopausal	4 (33.3%)
Size of lesion, cm, n (%)	
≤4	2 (16.7%)
>4	10 (83.3%)
FIGO stage, n (%)	
IB	2 (16.7%)
IIA	2 (16.7%)
IIB	3 (25.0%)
IIIA	3 (25.0%)
IIIB	1 (8.3%)
IVA	1 (8.3%)
Chemotherapy, n (%)	
Yes	10 (83.3%)
No	2 (16.7%)
The intent of chemotherapy, n (%)	
Neo-adjuvant	8 (80.0%)
adjuvant	2 (20.0%)
Protocol, n (%) (n=10)	
Carb/Pac	10 (100.0%)

Surgery was performed in four patients (33.3%), with FIGO stages distributed as follows: IIA in two patients (50%), IIB in one patient (25%), and IIIA in one patient (25%). Lymphovascular space invasion (LVSI) was identified in two (50%) of the surgical cases. As surgery was performed before presenting to the hospital, these patients were then staged and treated according to their pathological stage.

Induction chemotherapy was administered in eight cases (80%) before concurrent chemoradiation, while two patients (16.7%) received chemotherapy in an adjuvant setting after surgery. The median number of chemotherapy cycles received was three, ranging from zero to six cycles. A total of 11 patients (91.7%) who were treated with radical intent received radical concurrent chemoradiotherapy followed by brachytherapy, reflecting a standard approach for locally advanced cases. One patient received only radiotherapy with palliative intent (Table 2).

**Table 2:** Details of radiation therapy, concurrent chemotherapy, and brachytherapy

Radiation parameters			N (%)
Intent	Radical	11 (91.7%)	
	Palliative	1 (8.3%)	
Dose	45	3 (25.0%)	
	50.4	9 (75.0%)	
Fractions	15	1 (8.3%)	
	25	2 (16.7%)	
	28	9 (75.0%)	
Boost	Yes	1 (8.3%)	
	No	11 (91.7%)	
Concurrent chemotherapy	Yes	11 (91.7%)	
	No	1 (8.3%)	
Concurrent chemotherapy protocols	Cisplatin	10 (83.3%)	
	Carboplatin	1 (8.3%)	
Number of cycles	4.00	1 (8.3%)	
	5.00	9 (75.0%)	
	6.00	1 (8.3%)	
Brachytherapy	Yes	11 (91.7%)	
	No	1 (8.3%)	

**Figure 1:**
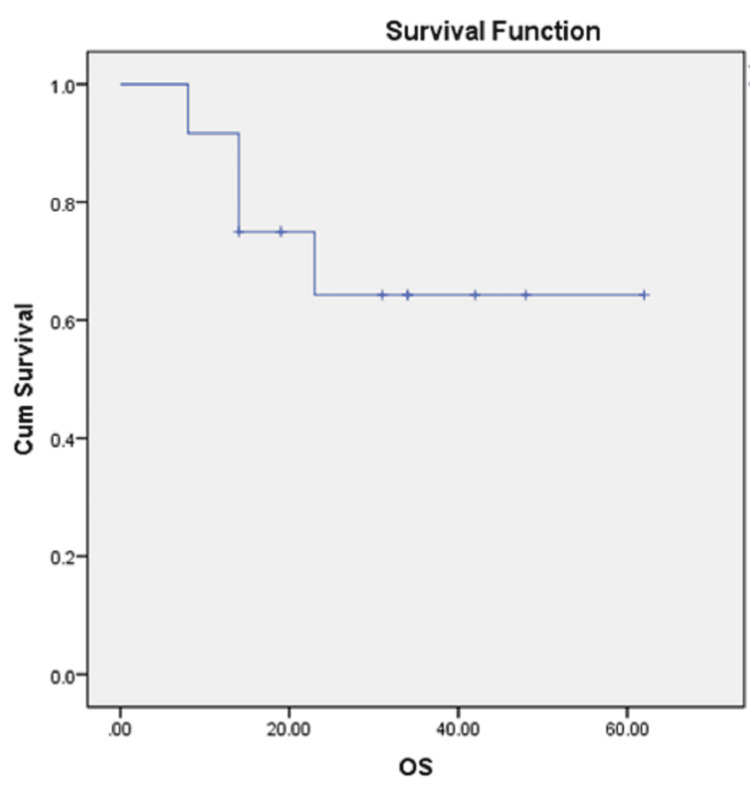
Overall survival curve OS: overall survival

The five-year overall survival in our study was 64.3% (Figure 1). During the study period, disease recurrence occurred in three patients (25%), with two patients developing distant metastasis and one developing locoregional recurrence.

## Discussion

CCAUC represents a small subset of cervical cancers and is particularly rare among patients without DES exposure. Our study reinforces the notion that this cancer is increasingly found in older women, contrary to its historical association with young women in the DES era [10]. Our findings align with those of Yang et al. [8] and Thomas et al. [7], who reported median ages of 52 and 53 years, respectively. Similarly, Reich et al. [1] reported a median age of 47 years in non-DES-exposed patients. The optimal treatment strategy for CCAUC remains unclear due to limited data. However, surgical management remains the mainstay for early-stage disease (FIGO I-II). Radical hysterectomy with lymphadenectomy is standard, and the National Comprehensive Cancer Network (NCCN) guidelines recommend surgery or radical radiotherapy as first-line treatments.

Studies by Irie et al. [11] and Baalbergen et al. [12] indicated better outcomes in adenocarcinoma patients treated with primary surgery compared to radiotherapy. Shimada et al. [13] reported higher recurrence rates in CCAUC patients treated with adjuvant radiotherapy compared to SCC (24.6% vs. 10.5%, p=0.0022). Nonetheless, patients with high-risk features may benefit from concurrent chemoradiation. In our cohort, 83.3% received chemotherapy, with a high rate (91.7%) of concurrent chemoradiation. These numbers reflect institutional treatment protocols based on patient stage and resectability [14]. Surgery was generally reserved for early-stage cases [15]. Tang et al. [16] highlighted the potential benefits of combining neoadjuvant chemotherapy (NACT) with chemoradiation in advanced adenocarcinomas. Our findings are consistent with these insights, with modest survival and recurrence rates. Despite chemotherapy, 33.3% of patients in our study died during the study period.

Despite offering valuable insights, our study is constrained by its retrospective design and limited sample size. As the study included patients treated with different approaches, an inference cannot be made on a standard approach for these patients based on this study. However, considering the extreme rarity of clear cell adenocarcinoma of the cervix in non-DES-exposed populations, conducting large prospective trials may not be feasible. The current literature largely consists of case reports and small retrospective series, which hinders the development of standardized treatment guidelines and limits the strength of outcome comparisons across studies.

## Conclusions

CCAUC is increasingly observed in older women in the post-DES era. While standard treatment guidelines are lacking, primary surgery remains the preferred approach in early-stage disease, supported by adjuvant radiotherapy and chemotherapy for high-risk cases. This retrospective analysis contributes valuable insights into clinical outcomes and emphasizes the need for larger, prospective studies to guide optimal treatment.
